# Guiding, sustaining and growing the public involvement of young people in an adolescent health research community of practice

**DOI:** 10.1111/hex.13616

**Published:** 2022-10-27

**Authors:** Teresa Swist, Philippa Collin, Betty Nguyen, Cristyn Davies, Patricia Cullen, Sharon Medlow, S. Rachel Skinner, Amanda Third, Katharine Steinbeck

**Affiliations:** ^1^ Institute for Culture and Society Western Sydney University Penrith New South Wales Australia; ^2^ Education Futures Studio, Sydney School of Education and Social Work University of Sydney Camperdown New South Wales Australia; ^3^ Young and Resilient Research Centre Western Sydney University Penrith New South Wales Australia; ^4^ Specialty of Child and Adolescent Health, Children's Hospital Westmead Clinical School, Faculty of Medicine and Health University of Sydney Westmead New South Wales Australia; ^5^ School of Population Health UNSW Sydney Kensington New South Wales Australia; ^6^ Ngarruwan Ngadju, First Peoples Health and Wellbeing Research Centre, Australian Health Services Research Institute University of Wollongong Wollongong New South Wales Australia; ^7^ The George Institute for Global Health UNSW Sydney Newtown New South Wales Australia; ^8^ Speciality of Child and Adolescent Health, Faculty of Medicine and Health, Sydney Medical School The University of Sydney Sydney New South Wales Australia; ^9^ Academic Department of Adolescent Medicine The Children's Hospital Westmead Westmead New South Wales Australia

**Keywords:** adolescent health, community of practice, co‐production, partnerships, public involvement in research, youth engagement

## Abstract

**Background:**

Public involvement in health research and its translation is well recognized to improve health interventions. However, this approach is insufficiently practised and evidenced in relation to young people. This paper presents an analysis of the process of co‐producing a framework, partnership model and a growing network of young people informing and guiding an adolescent health research community of practice.

**Methods:**

A Living Lab is a participatory research approach that brings together a broad range of stakeholders in iterative cycles of research, design, development, pilot‐testing, evaluation and delivery to implement effective responses to complex phenomena. The geographical setting for this study was Sydney, NSW, Australia, and involved both youth and adult stakeholders from this region. The study spanned three phases between July 2018 and January 2021, and data collection included a range of workshops, a roundtable discussion and an online survey.

**Results:**

The co‐production process resulted in three key outputs: first, an engagement framework to *guide* youth participation in health research; second, a partnership model to *sustain* youth and adult stakeholder collaboration; third, the *growth* of the public involvement of young people with a range of projects and partners.

**Conclusions:**

This study investigated the process of co‐producing knowledge with young people in an adolescent health community of practice. A reflexive process supported youth and adult stakeholders to collaboratively investigate, design and pilot‐test approaches that embed young people's engagement in adolescent health research. Shared values and iterative methods for co‐production can assist in advancing mutual learning, commitment and trust in specific adolescent health research contexts.

**Public Contribution:**

Young people guiding and informing an adolescent health research community of practice were involved in this study, and one of the participants is a paper co‐author.

## 1 INTRODUCTION

The principle of youth participation is well established and enshrined in international policy, including the World Health Organization Global Standards for Quality Healthcare Service for Adolescents,^1^ and the UN Convention on the Rights of the Child.^2^ Multistakeholder partnerships between organizations and young people can help embed meaningful engagement with young people across the health system, and progress health as one of the sustainable development goals.^3^, ^4^ Youth participation is increasingly recognized as fundamental to achieving effective adolescent health policy and services.^5^, ^6^, ^7^ While the benefits of youth participation in research and translation are considerable, realizing this benefit to adolescent health and well‐being remains a challenge. Greater collaborative practice, youth‐led approaches and adult stakeholder understanding and commitment are needed.^8^, ^9^, ^10^, ^11^, ^12^, ^13^ These require consideration of how existing institutions, processes and practices of research and translation that are adult‐centric can engage meaningfully with young people, their knowledge and needs.^14^, ^15^ Pragmatic ways to achieve ongoing youth engagement in multiple settings are under‐researched. Youth ‘consultations’ or advisory mechanisms are increasingly common.^16^, ^17^ Yet youth engagement in research too often commences after research questions, design and protocols are already defined, rather than involving young people from the beginning.

The expanding range of children's and young people's contributions to public involvement and engagement activities in health‐related research requires researchers to adopt pragmatic and flexible approaches which can ‘offer children and young people worthwhile ways of contributing to research with the level, purpose and impact of involvement determined by the children and young people themselves’.^18^,p.20 Instead of framing, and subsequently evaluating, public involvement in health research as an intervention, or output, there is increasing recognition of the value of continuous reflection, based on dialogue and learning between researchers and the public.^19^, ^20^ Such critical public involvement research seeks to explore the complexity of the relationship between researchers and the public, using methods to illuminate (and not simply measure) the complexity of dialogue.^19^,p.6

Ozer et al.^21^ argue that advancing youth participation in adolescent health research requires rigorous practice‐based evidence supported by ‘research‐practice partnerships’. Such partnerships are characterized by mutual learning, long‐term commitments and trust‐based relationships.^22^ Identification of approaches that facilitate a research‐practice partnership with young people is needed, to inform the much‐needed design, delivery and translation of adolescent health research. This partnership style first started in the education sector but has since broadened to areas such as child welfare and mental health.^23^, ^24^ In Australia, despite inclusion in policy commitments,^25^ there is currently no state or national mechanism to guide, sustain and grow, the public involvement of young people across adolescent health research, policy and practice.

To address this gap, the Well‐being Health & Youth Centre of Research Excellence (WH&Y CRE) identified the need to establish a way for young people to inform and guide adolescent health research and translation. WH&Y CRE is a multiuniversity research programme primarily funded by the National Health and Medical Research Council (NHMRC) and Western Sydney University. The WH&Y CRE brings together national expertise on adolescent health, ethics, digital media cultures, youth participation, epidemiology, health economics, health policy and practice development to research and inform adolescent healthcare and policy. The goal of this study was to document the process of guiding, sustaining and growing the public involvement of young people in an adolescent health research community of practice. Accompanying study objectives were to (i) investigate how to support youth participation across health research; (ii) Co‐design a model to enable youth and adult stakeholder collaboration and (iii) Pilot‐test the model to increase the public involvement of young people in an adolescent health research community of practice. These interrelated goals and objectives have led to the establishment of the Well‐being Health & Youth Commission (WH&Y Commission)^26^: a cross‐institutional commission of young people to collaborate with and advise researchers, policymakers and service providers in health research and research translation, through ethical engagement.

The process of expanding the public involvement of young people in an adolescent health research community of practice is the focus of this paper. To communicate a particularly complex, multiyear process, we document this study across three phases, with a concluding analysis that highlights how these phases interrelate with one another.

## 2 METHODS

### 2.1 Study setting, participants and recruitment

**Figure 1:**
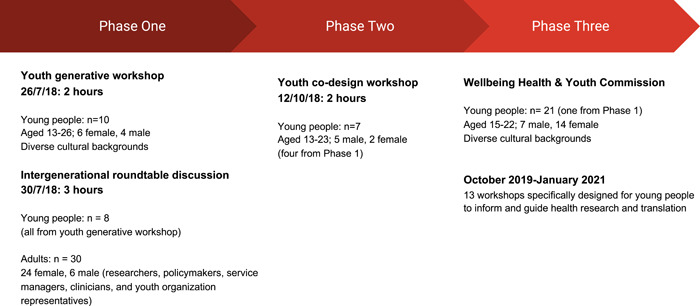
Study recruitment overview

This study was conducted in Sydney, the capital of New South Wales, Australia. Young people living in metropolitan Sydney, researchers and professionals working in adolescent health, policy and advocacy were invited to take part. Purposeful sampling was used to select youth participants who have interest, knowledge or lived experiences of health issues—particularly relevant to participatory research when the priorities and expertise of community partners are central to the study. Recruitment flyers were sent via email to the investigator's networks, noting that youth participants would be reimbursed with a gift voucher for their time. The purpose of restricting the target recruitment location was to trial the study in a location where youth participants could meet investigators in person at workshops. An overview of this study's recruitment is detailed across three study phases (Figure 1). Criteria for youth participants were that they reside in the greater metropolitan Sydney area, are interested in research and youth health, and are aged between 13 and 26 years. All recruitment information was in English, so the expectation was that young people were able to read, write and speak English. The study sought a diverse group with representation from young people with lived experience of health or mental health conditions, disability, migration, sexuality and gender diversity and from diverse social and cultural backgrounds because these young people are most likely to experience inequity in the health system in New South Wales. Additional participants for Phase 1 were adult researchers invited from the WH&Y CRE investigator network and policymakers and service providers. This study was approved by Western Sydney University Human Research Ethics Committee (Approval number: H11940).

### 2.2 Research design

**Figure 2:**
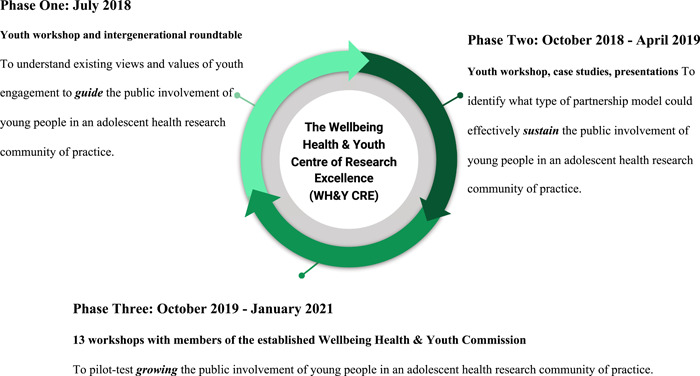
Study phase overview

The Living Lab methodology brings together a broad range of stakeholders in ongoing phases of co‐creation in real‐life settings and communities to implement effective responses to complex social and cultural phenomena.^27^ Living Labs create durable structures for integrating co‐research with co‐design over time.^28^, ^29^ Using qualitative methods of multiple workshops, a roundtable discussion and an online survey, young people and other stakeholders took part in an iterative process to identify and respond to key issues for youth engagement in adolescent health research.^30^ Our overall aim was to identify how to guide, sustain and grow the public involvement of young people in an adolescent health research community of practice—the WH&Y CRE. Between July 2018 and January 2021, iterative cycles of participatory research and design were undertaken (Figure 2). Supports available for young people for whom sensitive issues may have arisen, or vulnerabilities may have been present, included communicating workshop topics in advance so participants could decide if they want to attend (and, if so, prepare in advance), ensuring facilitation and activities offered various options for participation (such as deciding their preferred role across both whole group and small group discussions), alongside clear guidelines for ongoing feedback and support with the research team to address any concerns.

## 3 PHASE 1: TO UNDERSTAND EXISTING VIEWS AND VALUES OF YOUTH ENGAGEMENT TO *GUIDE* THE PUBLIC INVOLVEMENT OF YOUNG PEOPLE IN AN ADOLESCENT HEALTH RESEARCH COMMUNITY OF PRACTICE

### 3.1 Method

The first activity in this phase, a generative workshop, elicited young people's understandings and *values* that they thought would best support youth engagement in research and its translation. In small groups, participants brainstormed what they valued about four key topics related to adolescent health (based on the WH&Y CRE research programme): health, technology, research and youth participation. Ideas from each group were written on a large piece of paper and rotated around so that every group had a chance to add to, or expand upon, the insights. Participants in each group then synthesized the ideas aligned with a particular topic, so as to generate a ‘values statement’ that could be communicated to adult and other youth stakeholders. These statements substantively informed the second activity in this phase: an intergenerational roundtable discussion. The discussion was designed to elicit the experiences of young people in dialogue with other experts in a range of fields undertaking research, practice and policy for adolescent health and well‐being. To centre young people's values and voices in the discussion, three participants from the youth workshop were supported as co‐presenters, which involved informal mentoring from the research team to learn about the workshop process and skills required. They presented values statements from the youth workshop and were rapporteurs for the day. The small group discussion activity involved participants reflecting and building upon young people's value statements from the perspective of different stakeholder groups—young people, policymakers, researchers, health professionals and service representatives. These insights then informed the larger, whole group discussion that followed. A preliminary analysis of insights resulted in a draft framework, which was refined and developed with input from youth and adult stakeholders. A draft framework was discussed, revised and refined with key WH&Y investigators, commissioners and workshop participants.

### 3.2 Results

Phase 1 involved one youth workshop (*n* = 10; four males, six females) and one intergenerational roundtable (adults, *n* = 30; youth, *n* = 8). Attitudes towards and experiences of youth engagement in adolescent health research were identified from these activities and then grouped into three sets of values (Table 1) and their accompanying ethical practices. The resulting WH&Y Engagement Framework describes these findings for the three value sets alongside practical questions to prompt thought, reflection and action about ethical practices of engagement with young people (Table 1).^30^ This WH&Y Engagement Framework is represented in a publicly available document, with an accompanying infographic.^31^

**Table 1:** WH&Y Engagement Framework

First value set: Mutual trust and accountability
Questions
– Does the structure and governance of your work support young people's participation and contribution in meaningful ways? – Are there ongoing opportunities for young people to hear about progress and voice their ideas and concerns?
Ethical practices
– Producing a common language and meaningful technologies. – Actively engaging with all stakeholders to ensure the language used, activities planned, and technologies created are easy to understand, easy to join in with and make young people feel safe, comfortable and welcome.
Second value set: Diversity and Inclusion
Questions
– How can you best support young people and their networks in the co‐design of health research and translation? – Is your co‐design approach youth‐centred, strengths‐based and focused on maximizing opportunities for health and well‐being?
Ethical practices
– Co‐designing projects, systems and services. – Entering into engagement and collaboration with an open mind and understanding that young people's insights may test your thinking, challenge your assumptions and shift your goals.
Third value set: Equity and responsiveness
Questions
– In your communications are you using language, information and data that are inclusive, clear and understandable for a diversity of young people? – Are your material technologies (like consent forms) and social activities (like workshops) inclusive and respectful of young people's diverse, identities, abilities and skills?
Ethical practices
– Embedding a shared, intergenerational responsibility. – Developing collaborative processes that give stakeholders a sense of mutual ownership and shared responsibility and genuine opportunities to contribute and feedback.

Abbreviation: WH&Y, Well‐being Health & Youth.

The purpose of Phase 1 was to investigate the values that underpin youth participation across health research. Insights from a youth workshop and intergenerational roundtable informed the generation of the WH&Y Engagement Framework. The framework's value sets, questions and ethical practices guided the partnership model detailed in Phase 2.

## 4 PHASE 2: TO CO‐DESIGN A MODEL TO SUPPORT THE INVOLVEMENT OF YOUNG PEOPLE IN AN ADOLESCENT HEALTH RESEARCH COMMUNITY OF PRACTICE

### 4.1 Method

Over the course of a 2‐h workshop held in October 2018, youth participants generated ideas for a partnership model that could support young people's ongoing involvement in health research. First, fictional youth characters (known as ‘personas’ in design research) were discussed and developed to show the range of young people who might take part in the Commission (covering key aspects such as age, gender, location, technology use and background). Next, the relationship between these personas and the proposed Commission, known as ‘user journey’ in design research, was explored in response to six key questions: (i) How do they hear about the Commission? (ii) What would motivate them to get involved? (iii) How much time do they have to contribute? (iv) What types of events and activities would interest them? (v) What different types of incentives (e.g., reimbursement, recognition, skill‐building, networking) would keep them engaged? (vi) Why would you recommend the Commission to others? A draft model was discussed, revised ad refined with WH&Y investigators and commissioners to consolidate the model features.

### 4.2 Results

All the young people involved in Phase 1 were invited to participate in the Phase 2 workshop. Seven of those young people (five males and two females aged between 13 and 23 years) took part. The model generated (depicted in the right‐hand column of Table 2) describes the five main model features that were identified as central to sustaining an ongoing partnership between young people and researchers: structure and governance; membership; communication; recruitment and reimbursement; and activities. Participants also confirmed the value of calling the proposed model a ‘Commission’ as it signalled the important scope and profile of the proposed initiative—and distinguished it from advisory groups. Initially called the Adolescent Health Research Commission, it became the WH&Y Commission to reflect its alignment with the Centre of Research Excellence.

**Table 2:** Co‐produced framework and partnership model alignment

WH&Y CRE ethics of engagement (Phase 1)	WH&Y Commission partnership model features (Phase 2)
Does the structure and governance of your work support young people's participation and contribution in meaningful ways?	*Structure and governance*: The WH&Y CRE will provide organizational support and processes to enable knowledge sharing between the WH&Y Commission and diverse stakeholders from a variety of ages, backgrounds and sectors. This spans consultation and partnership in decision‐making, such as new research and funding proposals.
How can you best support young people and their networks in the co‐design of health research and translation?	*Membership*: The WH&Y Commission will offer a flexible range of ways for young people to be involved according to their interests and capacities (including a core group and a broader network). Participants will be provided with relevant training and have the opportunity to request or provide peer‐based learning on topics of interest.
In your communications are you using language, information and data that are inclusive, clear and understandable for a diversity of young people?	*Communication*: The WH&Y Commission will combine online and offline modes of communication to reflect the multiple places and times young people like to connect. Co‐creating and communicating outputs will support shared learning and capacity building between health experts, interdisciplinary researchers and young people.
Are there ongoing opportunities for young people to hear about progress and voice their ideas and concerns?	*Recruitment and reimbursement*: The WH&Y Commission will explore novel ways for recruiting a diversity of young people and representation from marginalized or excluded groups. Reimbursement processes, the contribution of members and research impact over time, will all be clearly communicated.
Are your material technologies and social activities inclusive and respectful of young people's diverse, identities, abilities and skills?	*Activities*: The WH&Y Commission will offer a variety of inclusive and fun activities which enhance the capacities of young people and make a meaningful contribution to research, policy and practice. Online and in person activities will engage with young people's interests, support networks and organizations to maximize opportunities for impactful health and well‐being initiatives. Collaborative approaches to research design, delivery and translation will be prioritized.
Is your co‐design approach youth‐centred, strengths‐based and focused on maximizing opportunities for health and well‐being?	

Abbreviation: WH&Y CRE, Well‐being Health & Youth Centre of Research Excellence.

To demonstrate the interrelationship between the first and second research phases, we aligned the guiding questions from the Engagement framework with the identified partnership model features (Table 2).

This partnership model was further informed by five case studies of successful youth engagement approaches of health and youth‐related organizations in Australia (Canteen, Headspace, Multicultural Youth Action Network, Youth Action and Youth off the Streets). These case studies identified the importance of clear aims, structure, targeted recruitment and communication strategies—across a range of national, regional and local level entities. These examples of organizational best practices helped refine and validate the partnership model.

The model was presented and revised at a series of academic, youth sector and health professional events to review and refine the proposed model. These events took place between 2018 and 2019 and included: the Australasian Association of Adolescent Health Conference; the University of Sydney Public Involvement in Health Research; a WH&Y CRE investigators meeting; and a New South Wales Ministry of Health One Day Youth Health Showcase. Based on feedback at these presentations, specific research and policy use cases were generated to show how this partnership model could work in practice with various stakeholders.

The purpose of Phase 2 was to co‐design a partnership model to sustain youth and adult stakeholder collaboration in alignment with the WH&Y Engagement Framework. A youth co‐design workshop and five cases of health and youth‐related organizations informed the partnership model which became the foundation for implementing Phase 3 across five key areas: recruitment and reimbursement; communication; structure and governance; activities and membership.

## 5 PHASE 3: TO PILOT‐TEST *GROWING* THE PUBLIC INVOLVEMENT OF YOUNG PEOPLE IN AN ADOLESCENT HEALTH RESEARCH COMMUNITY OF PRACTICE

### 5.1 Method

This phase focused on the formal recruitment and pilot of the WH&Y Commission. A call for expressions of interest was circulated to young people involved in previous phases, via researcher networks, and on social media. Prospective applicants were invited to write up to 500 words to (i) outline health issues they were interested in; (ii) why they wanted to be involved in the WH&Y Commission, such as lived experiences; (iii) the skills and knowledge they could bring and (iv) health topics they were passionate about (the responses are summarized in Table 3). Iteratively informed by the framework and model developed in previous phases, the WH&Y CRE researchers facilitated a series of small‐scale activities with appointed Commissioners to pilot‐test how the WH&Y Commission could advance youth engagement in adolescent health research, policy and advocacy. Between October 2019 and January 2021, the team facilitated thirteen 2–3 h workshops with the Commissioners to pilot‐test growing the public involvement of young people in an adolescent health research community of practice. Activities were conducted as in‐person events until the pandemic necessitated online events (via Zoom). Activities were designed to increase the knowledge and skills of young people and researchers to work together, and enable young people's perspectives to guide and co‐create youth health research (detailed further in Section 5.2). A draft model was discussed, revised and refined with WH&Y investigators and Commissioners to ensure that the dimensions and details authentically reflected key insights from the data collection process.

**Table 3:** Recruitment questionnaire overview

Question	Responses summary
What health issues are you interested in?	− Well‐being, anxiety and depression, plus mental health (including culturally and linguistically diverse communities, students, the medical profession, young people in rural areas) − Public health (such as vaccination), rural health, health over the life‐course − LGBTQI + youth, sexual and reproductive health, sexual health and safety, plus same‐sex sexual health education classes in schools and communities − Nutrition and fitness, childhood obesity, physical inactivity − Health literacy and education, health equity/inequities, health access and experiences (young women, culturally diverse young men, Indigenous youth, migrant and refugee youth) − Alcohol and drug abuse, substance abuse − Disability, heart disease, cancer, families affected by cancer, cardiometabolic health and diabetes − Interrelationship between mental health and physical health, domestic violence
Why do you want to be involved in the WH&Y Commission?	− Share personal and lived experiences (such as marginalization, low socioeconomic backgrounds, mental health issues) − Their connection to living in Western Sydney (their local community) − Raise awareness, further education, to empower others, making change for all youth (particularly those who do not have a voice), to advocate for youth in schools, create and establish a meaningful voice for youth
What are some of your skills &/or experiences that you would be able to add to the WH&Y Commission?	− Connections to existing youth network and advocacy groups, previous/existing volunteering, committee experience, youth advisory and project experience − Interpersonal skills, leadership and teamwork, online/in‐person communication, administration, health services knowledge, critical thinking and research skills − Public speaking, event management, leadership, urban planning, creativity and imagination, health promotion, photography, film and graphic design
What are some topics you are passionate about that you would like to bring to your work in the WH&Y Commission?	− Inclusion, equality, diversity, migrant and refugee youth − Important role of education and employment − The role of technology (such as moving away from Dr Google, increasing accessibility of information and services to young people) − The future of cities and shaping a more health resilient city, urban planning and resilience − Feminism, multiculturalism − Discrimination, stigma and cultural taboos − Community engagement and leadership

Abbreviation: WH&Y CRE, Well‐being Health & Youth Centre of Research Excellence.

### 5.2 Results

The results for Phase 3 are aligned with key dimensions of our partnership model features to demonstrate how previous research phases informed this phase.

#### 5.2.1 Recruitment and reimbursement

Twenty‐one young people aged 15–22 years old responded to the recruitment call (7 males and 14 females) and all were invited to join the WH&Y Commission. Eight young people were recruited through youth‐serving organizations that represented local health services, mental health or multicultural youth. One member specified that their interest in the initiative was piqued by the recruitment call‐out communicated via the Instagram account. All participants lived in metropolitan Sydney and had diverse cultural backgrounds and lived experiences. Remuneration and recognition for Commissioners included 100 AUD for participation in monthly workshops, informal mentoring and letters of appreciation. For example, letters of appreciation were sent from the WH&Y CRE's Chief Investigator to all Commissioners at the end of 2020 to communicate thanks and recognize their contributions over the course of the year.

#### 5.2.2 Communication

Initial in‐person workshops, WH&Y Commission activities and expectations were rapidly adapted during the COVID‐19 pandemic which forced researchers, young people and policymakers into immediate and unexpected priorities and modes of work, particularly online. With government lockdowns and limited numbers for in‐person events in place, we replaced our regular workshops with online Zoom meetings supplemented with a Facebook group. Also, we were mindful of the extra stress young people experienced during the crisis, so ensured that any planned activities and timelines did not place any additional pressure upon Commissioners.

#### 5.2.3 Structure and governance

The WH&Y Commission is structured as a collective, with a coordinator who supports the activities of the group. WH&Y CRE investigators, partners and other stakeholders can make requests to work with the WH&Y Commission which are then vetted by the CRE Chief Investigators and the Commissioners themselves. Commissioners attend monthly workshops, communicate online and can opt‐in to work on specific projects and activities. Activities are organized around four pillars: *Build Capacity; Advance Research; Advise and Co‐Create*.

#### 5.2.4 Activities

This pilot‐testing phase has demonstrated growing engagement between the WH&Y Commission and a range of research, policy and practice initiatives. Commissioners contributed to research priority setting, design and development aligned across the following areas:

(1) *Build capacity*: The WH&Y Commission has facilitated skill and knowledge building among young people and researchers. Researchers and policymakers have gained a new understanding of youth perspectives and how youth participation can be a part of youth health research. WH&Y Commissions have received training in research methods, ethics, communication and blog writing, as well as topic areas such as gender and sexuality.
(2) *Advance research*: Commissioners have advanced the focus of interdisciplinary health research. This included identifying key issues for youth health during COVID‐19; providing feedback on the design and ethics of a virtual reality research project to reduce stress in young people waiting in the emergency room; providing input to grant submissions; contributing key insights for emerging technologies literature review; and co‐creating future healthcare scenarios to inform research priority‐setting.
(3) *Advise and co‐create*: The WH&Y Commission is meeting the demand for youth advisors and co‐creators across health research, policy and translation. Commissioners have informed policy across regional, national and international settings. For instance, 15 Commissioners were involved in assessing existing and generating new indicators of youth health for a federal government report^32^; 2 Commissioners were appointed to the New South Wales Ministry of Health Youth Advisory Board, providing peer research and advice on young people's healthcare needs during the initial phases of the COVID‐19 pandemic; 4 Commissioners contributed to a national youth health leaders forum led by Australia's Consumer Health Forum generating a report on priorities for youth health for the Federal Minister of Health and Youth^33^; 5 Commissioners contributed to a range of national and international policy submissions including a response to the UNCRC Draft General Comment on the Rights of the Child in the digital environment.^34^ Commissioners advocate for health issues and help broaden project audiences through blog‐writing, co‐authoring publications and curating social media content. At the end of 2020, an overview of WH&Y Commission activities was reported.^35^

#### 5.2.5 Membership

In February 2020, Commissioners were invited to evaluate their participation in the first 3 months. Fourteen of the Commissioners responded to the online survey (Table 4).

**Table 4:** WH&Y Commission survey questions and responses

Questions	Responses
Benefits of involvement	Meeting like‐minded people, learning about youth health perspectives in Australia; building confidence, communication and networking skills; learning about challenges young people face.
How they felt about communicating their involvement	Approximately 85% of respondents said they were confident when talking about the WH&Y Commission and its role, while the remainder wanted to be provided with more information such as objectives and timelines.
New skills they wanted to learn	Advocacy and policy, media training, mental health and sexual health issues, public health issues and public speaking.
Workshop feedback	Online commentary during face‐to‐face workshops, using Prezzee^R^ platform, was viewed positively by all respondents; adding different ice‐breakers and team bonding activities.
Reasons for continuing as a member	To learn more about youth health, health advocacy, ensure youth voices are being heard, ability to step outside one's comfort zone.

Abbreviation: WH&Y, Well‐being Health & Youth.

In December 2020, all Commissioners were invited to complete a short survey about their involvement including what worked well, achievements, experiences, ideas for improvement, interest in continuing and any additional reflections. Two‐thirds of Commissioners responded (*n* = 13) and survey results identified that: Commissioners overwhelmingly valued having a platform from which to learn about and share their views on what adolescent health research should achieve; to advocate on specific topics, collaborate and be heard by researchers and policymakers; and to learn new skills. They wanted to play a greater role in leading the discussion and setting priorities in adolescent health research and service delivery.

All WH&Y Commission members—including youth participants from Phase 1—have had opportunities to extend and adapt their involvement according to their interests and needs. For example, a Phase 1 participant became a research assistant and co‐facilitator of the WH&Y Commission, and now a co‐author of this paper (B. N.). Another inaugural member is co‐authoring a research paper related to the healthcare scenarios workshops. A minimum number of attendances at the monthly workshops is not mandated, as we recognize young peoples' multiple commitments and priorities (alongside the additional pressures of COVID‐19). Nevertheless, participation in workshops was at least 50% each workshop over the course of 2020 and 60% of Commissioners in 2020 opted to renew their membership in the group in 2021. The partnership model co‐produced with young people specified a core membership group of young people who are consistently collaborating with researchers, *in addition to* a broader network of young people that includes: peers of current Commissioners, past Commissioners, prospective Commissioners (who have expressed interest), alongside dual membership of other policy and practice groups (such as the Consumer Health Forum).

The purpose of Phase 3 was to pilot‐test growing the public involvement of young people in an adolescent health research community of practice. The team facilitated 13 workshops with the Commissioners over the course of 16 months. The framework questions (Phase 1) and partnership model features (Phase 2) enabled the team to examine the possibilities and limits, of how this Commission could be guided, sustained and grow in the future.

## 6 DISCUSSION

Our study contributes new knowledge about how the public involvement of young people in health research can be enabled from the perspective of an adolescent health research community of practice. This is distinct from involving young people in individual research projects and is aimed at understanding how to embed engagement with young people in ongoing processes of health research and translation. Lessons learned are aligned with three key research‐practice partnership principles: mutual learning, trusting relationships and long‐term collaboration.^36^ We have shown first that *mutual learning* between young people and adult stakeholders can be guided throughout a project with participatory methods that co‐produce a shared language and knowledge exchange within specific contexts. Next, that to sustain *trusting relationships* requires transparency and flexibility based on a co‐designed partnership model so that clear expectations can scaffold present and future collaborations. Finally, that *longer‐term commitments* with young people are vital to support health research capacity‐building and priority‐setting beyond short‐term funding cycles. These learnings correspond with calls for ongoing youth‐adult partnership research to focus on factors for success and adaptability to local contexts.^37^ We, therefore, seek to inspire national and international research programmes to discover how to guide, sustain and grow the public involvement of young people tailored to their specific contexts.

The strengths of our research emerged from expanding the public involvement of young people in health research with a Living Lab approach. This strengths‐based, iterative method generated opportunities for young people to work with, advise and collaborate with researchers, policymakers and peak bodies (an association of organizations with allied interests) despite the significant disruption caused by the COVID‐19 pandemic. This approach offered a way to prioritize and develop research outcomes, as well as understand processes of health research, and their impacts, in a multidisciplinary context involving youth and adult stakeholders. In addition, recruiting through networks and via existing youth‐serving organizations helped to recruit young people who may experience exclusion or disadvantage within the health system. One limitation to date is that the views of adult stakeholders about these initial phases are yet to be examined. This knowledge is important to understand the experiences, barriers and capacity development necessary for meaningful engagement of both youth and adult stakeholders.^38^

As reported, interest in working with the WH&Y Commission has been primarily from the advocacy and policy sectors. Our next focus is to continue to identify researchers to collaborate in the co‐creation of health research agendas, and design of research projects and enable more youth‐led initiatives to emerge from within research programme activities. Several grant applications have been submitted where WH&Y CRE collaborators have budgeted to involve the Commission and generate future methods, resources and tools to support researchers to engage with young people in health research. We acknowledge that the WH&Y Commission membership has been limited to metropolitan Sydney and there are plans to expand the initiative to other regions and states of Australia. Another limitation, due to the current size and geographic scope of the Commission, was that the range of lived experiences of the current group does not include young people living in remote, regional areas, indigenous young people or enough young people living with a disability or chronic health conditions. We acknowledge that this wider range of participants would require extra resourcing and support, which would be addressed in the process of scaling up the Commission in terms of Commissioners selected, as well as partner organizations.

Future research directions include the definition of the optimal ways for the WH&Y Commission to work with researchers within complex traditional research project cycles, where priorities are often set by funders and not by researchers or young people, and where short application deadlines render meaningful collaboration more challenging. Different funding models will also be explored to identify alternate avenues for expansion—as this style of research‐practice partnership requires significant investment, time, infrastructure, leadership and expertise.^39^ Sustaining and growing a research‐practice partnership with young people requires communicating the time and resources required, as well as the impact and value of youth participation to funding and grant bodies, researchers and other adult stakeholders. In Australia, national funding bodies do not currently prioritize research priority‐setting with young people and families, so this would be a unique innovation for the Australian research landscape. At the city scale, a current collaboration with the Sydney Children's Hospital Network Foundation is seeking funding to enable young people to contribute to priority‐setting. The WH&Y Commission has the potential to contribute much more to adolescent health research agendas and design and overcome additional barriers to youth participation in research across urban, regional and national scales. For example, to lead innovative ethics processes which, when balanced with necessary protections, advance and scale young people's engagement beyond traditional research ‘participant’ roles. We also plan to develop the digital infrastructure and participation literacies required to extend our WH&Y Commission to other urban and regional areas, so as to expand the ‘networked’ capabilities of our community of practice in relation to diverse people, places and platforms.^40^ This vision could be implemented by linking with other organizations and research projects—both in Australia and overseas—so as to advance the public involvement of adolescents across health research, policy and practice.

## 7 CONCLUSION

The project process documented in this paper was a complex, multiyear study about co‐researching and designing a youth‐engaged adolescent health research community of practice.

Crucial learnings included (i) understanding existing views and values of youth engagement to *guide* the public involvement of adolescents in an adolescent health research community of practice; (ii) identifying what type of partnership model could effectively *sustain* the public involvement of adolescents in an adolescent health research community of practice and (iii) pilot‐testing the *growth* of public involvement of adolescents in an adolescent health research community of practice. These insights contribute to literature focused on the public involvement of young people in health research, the acknowledged importance of reflexive and iterative process evaluations—plus the shared knowledge and value gained from co‐producing health research with young people in specific contexts. In sum, the study demonstrated the importance of the following research‐practice partnership principles: *mutual learning*, through co‐producing shared values; *trusting relationships*, with an intergenerational partnership; and, *long‐term collaboration*, based upon an ongoing conversation to expand the public involvement of young people in an adolescent health research community of practice.

## CONLICT OF INTEREST

The authors have no conflicts of interest to declare.

## Data Availability

Research data are not shared.
